# 3-Amino-5-(piperidin-1-yl)thio­phene-2,4-dicarbonitrile

**DOI:** 10.1107/S1600536811052950

**Published:** 2011-12-17

**Authors:** Wedad M. Al-Adiwish, D. Adan, Mohamed Ibrahim Mohamed Tahir, W.A. Yaacob, Mohammad B. Kassim

**Affiliations:** a School of Chemical Sciences and Food Technology, Faculty of Science and Technology, Universiti Kebangsaan Malaysia, 43600 Selangor, Malaysia; b Department of Chemistry, Faculty of Science, Universiti Putra Malaysia, 43400 UPM Serdang, Selangor, Malaysia; c Fuel Cell Institute, Universiti Kebangsaan Malaysia, 43600 Selangor, Malaysia

## Abstract

In the title compound, C_11_H_12_N_4_S, the thio­phene ring is roughly planar, with a maximum deviation of 0.012 (1) Å for the S atom, and makes a dihedral angle of 7.89 (8)° with the mean plane of the piperidine ring, which is in a chair conformation. The crystal packing is stabilized by pairs of centrosymmetric inter­molecular N—H⋯N hydrogen bonds, which results in the formation of a step-wise chain parallel to [10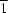].

## Related literature

For the biological activity of amino­thio­phene derivatives, see: Abdel-Fattah *et al.* (2006 ▶). For related structures, see: El-Saghier (2002 ▶); Eller & Holzer (2006 ▶); Thomae *et al.* (2009 ▶); Al-Adiwish *et al.* (2011 ▶). For standard bond lengths, see: Allen *et al.* (1987 ▶).
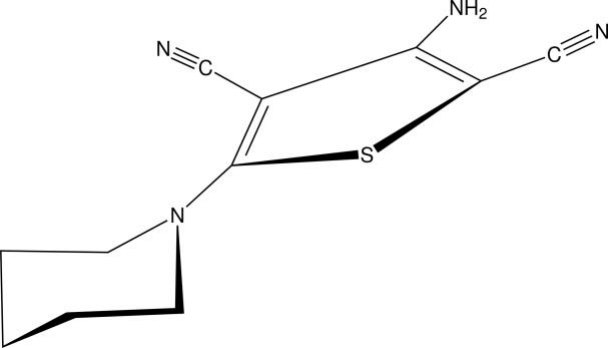


## Experimental

### 

#### Crystal data

C_11_H_12_N_4_S
*M* *_r_* = 232.31
Monoclinic, 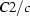

*a* = 14.1637 (3) Å
*b* = 11.2823 (3) Å
*c* = 14.4413 (3) Å
β = 98.131 (2)°
*V* = 2284.51 (9) Å^3^
*Z* = 8
Cu *K*α radiation
μ = 2.33 mm^−1^
*T* = 423 K
0.18 × 0.14 × 0.11 mm

#### Data collection

Oxford Diffraction Gemini CCD area-detector’ diffractometer
Absorption correction: multi-scan (*CrysAlis PRO*; Oxford Diffraction, 2006 ▶) *T*_min_ = 0.679, *T*_max_ = 0.784
11636 measured reflections
2188 independent reflections
2026 reflections with *I* > 2σ(*I*)
*R*_int_ = 0.024

#### Refinement

*R*[*F* ^2^ > 2σ(*F* ^2^)] = 0.035
*wR*(*F* ^2^) = 0.098
*S* = 1.05
2188 reflections
146 parameters
H-atom parameters constrained
Δρ_max_ = 0.36 e Å^−3^
Δρ_min_ = −0.29 e Å^−3^

### 

Data collection: *CrysAlis CCD* (Oxford Diffraction, 2006 ▶); cell refinement: *CrysAlis CCD*; data reduction: *CrysAlis RED* (Oxford Diffraction, 2006 ▶); program(s) used to solve structure: *SHELXS97* (Sheldrick, 2008 ▶); program(s) used to refine structure: *SHELXL97* (Sheldrick, 2008 ▶); molecular graphics: *SHELXTL* (Sheldrick, 2008 ▶); software used to prepare material for publication: *SHELXTL*, *PLATON* (Spek, 2009 ▶) and *publCIF* (Westrip, 2010 ▶).

## Supplementary Material

Crystal structure: contains datablock(s) I, global. DOI: 10.1107/S1600536811052950/kp2376sup1.cif

Structure factors: contains datablock(s) I. DOI: 10.1107/S1600536811052950/kp2376Isup2.hkl

Supplementary material file. DOI: 10.1107/S1600536811052950/kp2376Isup3.cml

Additional supplementary materials:  crystallographic information; 3D view; checkCIF report

## Figures and Tables

**Table 1:** Hydrogen-bond geometry (Å, °)

*D*—H⋯*A*	*D*—H	H⋯*A*	*D*⋯*A*	*D*—H⋯*A*
N1—H1*A*⋯N3^i^	0.86	2.22	3.0576 (19)	164
N1—H1*B*⋯N2^ii^	0.86	2.29	3.0293 (17)	145

Symmetry codes: (i) 
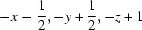
; (ii) 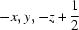.

**Fig. 1.**
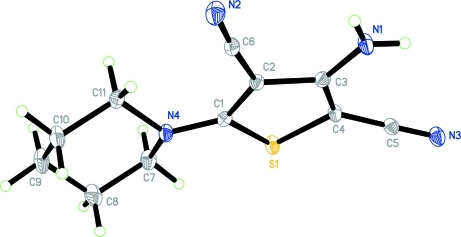
The molecular structure of (I) with displacement ellipsoids drawn at the 30% probability level.

**Fig. 2.**
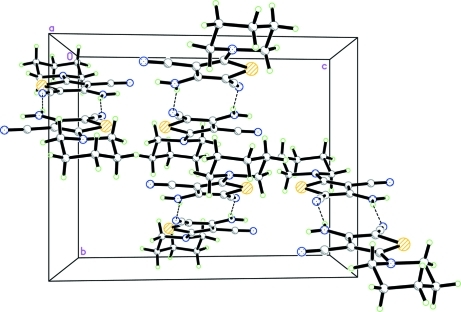
Crystal packing of (I) viewed down the a-axis. Hydrogen bonds are drawn as dashed lines.

## supplementary crystallographic information

### Comment

Recent studies have shown that aminothiophene derivatives are potential antibacterial and antifungal substances [Abdel-Fattah *et al.* (2006)]. The title compound (I), is a derivative of piperidine containing aminothiophene, was reported earlier [Al-Adiwish *et al.* (2011); Eller & Holzer (2006);  El-Saghier *et al.* (2002)]. However, its crystal structure is reported here. The thiophene fragment (S1/N1/N2/N4/C1/C2/C3/C4/C6) is close to be planar with the largest deviation of 0.034 (1)Å for S1. The two C(*sp*^2^)–C(*sp*^2^) bond lengths in the carbonitrile fragments differ slightly with C2–C6 1.417 (2)Å and C4–C5 1.403 (2) Å, respectively. Electron donation of the S atom may contribute to the increase in the former bond length. Angles in the respective fragments, C6–C2–C3 [118.50 (13) °] and C3–C4–C5 [125.50 (13) °]are different from the value typical for this hybridisation. Other bond lengths and angles in the molecule are in the normal ranges (Allen *et al.*, 1987).

In the crystal molecules are linked by two intermolecular N1–H1A···N2 and N1–H1B···N3 hydrogen bonds forming a centro-symmetric dimers along the crystallographic (010) direction (Table 1, Fig. 2).The intramolecular contact C-H···S between the pyperidine and thiophene rings was observed (Table 1).

### Experimental

The title compound was prepared according to previously report (Thomae *et al.*, 2009) with some modifications. 2-[Bis(methylthio)methylene]propanedinitrile (0.01 mol) was dissolved in DMF (15 mL) prior to addition of piperidine (0.01 mol). The mixture was heated at 343 K for 75 min and then Na_2_S.9H_2_O (0.01 mol) was added and heated for another 2 h. Then, chloroacetonitrile (0.02 mol) was added slowly and was left at 343 K for another 2 h. Subsequently, potassium carbonate (0.02 mol) was added and left for another 90 min. Finally, the reaction mixture was poured into water (100 mL) and stirred vigorously to give a white precipitate. The residue was filtered, washed with water, and dried at room temperature until a constant weight. A slow evaporation of the compound from methanol solution gave single crystals suitable for X-ray diffraction (yield = 74.0%).

### Refinement

The H atoms of both C and N atoms were positioned geometrically and allowed to ride on their parent atoms, with *U*_iso_ = 1.2*U*_eq_ (C) for CH_2_ 0.97 Å and *U*_iso_(H) = 1.2*U*_eq_(N) for N–H 0.86 Å.

### Crystal data

**Table:**

C_11_H_12_N_4_S	*F*(000) = 976
*M**_r_* = 232.31	*D*_x_ = 1.351 Mg m^−^^3^
Monoclinic, *C*2/*c*	Cu *K*α radiation, λ = 1.54178 Å
Hall symbol: -C 2yc	Cell parameters from 1189 reflections
*a* = 14.1637 (3) Å	θ = 3–71°
*b* = 11.2823 (3) Å	µ = 2.33 mm^−^^1^
*c* = 14.4413 (3) Å	*T* = 423 K
β = 98.131 (2)°	Plate-like, brown
*V* = 2284.51 (9) Å^3^	0.18 × 0.14 × 0.11 mm
*Z* = 8	

### Data collection

**Table:**

Oxford Diffraction Gemini CCD area-detector' diffractometer	2188 independent reflections
Radiation source: fine-focus sealed tube	2026 reflections with *I* > 2σ(*I*)
graphite	*R*_int_ = 0.024
ω/2θ scans	θ_max_ = 71.2°, θ_min_ = 5.0°
Absorption correction: multi-scan (*CrysAlis PRO*; Oxford Diffraction, 2006)	*h* = −15→17
*T*_min_ = 0.679, *T*_max_ = 0.784	*k* = −12→13
11636 measured reflections	*l* = −17→17

### Refinement

**Table:**

Refinement on *F*^2^	Secondary atom site location: difference Fourier map
Least-squares matrix: full	Hydrogen site location: inferred from neighbouring sites
*R*[*F*^2^ > 2σ(*F*^2^)] = 0.035	H-atom parameters constrained
*wR*(*F*^2^) = 0.098	*w* = 1/[σ^2^(*F*_o_^2^) + (0.0635*P*)^2^ + 1.3435*P*] where *P* = (*F*_o_^2^ + 2*F*_c_^2^)/3
*S* = 1.05	(Δ/σ)_max_ = 0.001
2188 reflections	Δρ_max_ = 0.36 e Å^−^^3^
146 parameters	Δρ_min_ = −0.29 e Å^−^^3^
0 restraints	Extinction correction: *SHELXL97* (Sheldrick, 2008), Fc^*^=kFc[1+0.001xFc^2^λ^3^/sin(2θ)]^-1/4^
Primary atom site location: structure-invariant direct methods	Extinction coefficient: 0.00105 (16)

### Special details

**Table:**

Experimental. The crystal was placed in the cold stream of an Oxford Cryosystems open-flow nitrogen cryostat (Cosier & Glazer, 1986) with a nominal stability of 0.1 K.Cosier, J. & Glazer, A.*M*., 1986. *J. Appl. Cryst.*105 107.
Geometry. All e.s.d.'s (except the e.s.d. in the dihedral angle between two l.s. planes) are estimated using the full covariance matrix. The cell e.s.d.'s are taken into account individually in the estimation of e.s.d.'s in distances, angles and torsion angles; correlations between e.s.d.'s in cell parameters are only used when they are defined by crystal symmetry. An approximate (isotropic) treatment of cell e.s.d.'s is used for estimating e.s.d.'s involving l.s. planes.
Refinement. Refinement of *F*^2^ against ALL reflections. The weighted *R*-factor *wR* and goodness of fit *S* are based on *F*^2^, conventional *R*-factors *R* are based on *F*, with *F* set to zero for negative *F*^2^. The threshold expression of *F*^2^ > σ(*F*^2^) is used only for calculating *R*-factors(gt) *etc*. and is not relevant to the choice of reflections for refinement. *R*-factors based on *F*^2^ are statistically about twice as large as those based on *F*, and *R*- factors based on ALL data will be even larger.

### Fractional atomic coordinates and isotropic or equivalent isotropic displacement parameters (Å^2^)

**Table:**

	*x*	*y*	*z*	*U*_iso_*/*U*_eq_	
S1	0.05434 (2)	0.13968 (4)	0.64677 (2)	0.02482 (16)	
N1	−0.09012 (9)	0.19216 (12)	0.39813 (8)	0.0267 (3)	
H1A	−0.1472	0.2138	0.4039	0.032*	
H1B	−0.0730	0.1865	0.3435	0.032*	
N2	0.11711 (10)	0.11912 (13)	0.30576 (9)	0.0324 (3)	
N3	−0.21230 (9)	0.19501 (13)	0.61321 (9)	0.0305 (3)	
N4	0.21438 (8)	0.07773 (11)	0.58195 (8)	0.0227 (3)	
C1	0.12328 (10)	0.11239 (13)	0.55838 (10)	0.0202 (3)	
C2	0.06959 (10)	0.13264 (12)	0.47006 (10)	0.0193 (3)	
C3	−0.02745 (10)	0.16710 (12)	0.47476 (10)	0.0201 (3)	
C4	−0.04625 (10)	0.17282 (14)	0.56599 (10)	0.0225 (3)	
C5	−0.13693 (10)	0.18534 (13)	0.59338 (9)	0.0229 (3)	
C6	0.09988 (10)	0.12325 (13)	0.38096 (10)	0.0224 (3)	
C7	0.25656 (11)	0.08466 (16)	0.68138 (10)	0.0294 (4)	
H7A	0.2077	0.0677	0.7203	0.035*	
H7B	0.2794	0.1646	0.6954	0.035*	
C8	0.33845 (12)	−0.00184 (17)	0.70481 (11)	0.0363 (4)	
H8A	0.3139	−0.0822	0.7006	0.044*	
H8B	0.3685	0.0114	0.7686	0.044*	
C9	0.41251 (11)	0.01221 (18)	0.63870 (12)	0.0394 (4)	
H9A	0.4414	0.0902	0.6464	0.047*	
H9B	0.4624	−0.0466	0.6531	0.047*	
C10	0.36437 (11)	−0.00355 (16)	0.53838 (12)	0.0325 (4)	
H10A	0.4110	0.0086	0.4961	0.039*	
H10B	0.3407	−0.0841	0.5299	0.039*	
C11	0.28218 (10)	0.08241 (15)	0.51370 (11)	0.0269 (3)	
H11A	0.3071	0.1623	0.5116	0.032*	
H11B	0.2490	0.0634	0.4520	0.032*	

### Atomic displacement parameters (Å^2^)

**Table:**

	*U*^11^	*U*^22^	*U*^33^	*U*^12^	*U*^13^	*U*^23^
S1	0.0192 (2)	0.0380 (3)	0.0174 (2)	0.00484 (13)	0.00300 (14)	−0.00005 (13)
N1	0.0193 (6)	0.0404 (8)	0.0200 (6)	0.0078 (5)	0.0022 (5)	−0.0001 (5)
N2	0.0253 (7)	0.0511 (9)	0.0213 (7)	0.0034 (6)	0.0055 (5)	0.0012 (6)
N3	0.0237 (7)	0.0427 (8)	0.0257 (7)	0.0076 (6)	0.0057 (5)	0.0045 (6)
N4	0.0163 (6)	0.0318 (7)	0.0196 (6)	0.0015 (5)	0.0017 (5)	0.0014 (5)
C1	0.0192 (7)	0.0209 (7)	0.0211 (7)	−0.0018 (5)	0.0048 (5)	0.0001 (5)
C2	0.0181 (7)	0.0213 (7)	0.0188 (7)	−0.0003 (5)	0.0036 (5)	0.0001 (5)
C3	0.0192 (7)	0.0197 (7)	0.0214 (7)	−0.0006 (5)	0.0030 (5)	−0.0004 (5)
C4	0.0175 (7)	0.0283 (8)	0.0213 (7)	0.0031 (6)	0.0017 (5)	−0.0005 (6)
C5	0.0232 (8)	0.0263 (8)	0.0189 (7)	0.0041 (6)	0.0017 (6)	0.0010 (6)
C6	0.0171 (7)	0.0256 (8)	0.0240 (8)	0.0010 (5)	0.0011 (6)	0.0014 (6)
C7	0.0233 (7)	0.0427 (10)	0.0212 (7)	0.0020 (6)	−0.0002 (6)	−0.0022 (6)
C8	0.0271 (8)	0.0510 (11)	0.0277 (8)	0.0070 (7)	−0.0065 (7)	0.0017 (7)
C9	0.0192 (8)	0.0573 (12)	0.0395 (10)	0.0081 (7)	−0.0035 (7)	−0.0037 (8)
C10	0.0211 (7)	0.0432 (10)	0.0328 (9)	0.0077 (7)	0.0030 (6)	−0.0009 (7)
C11	0.0198 (7)	0.0339 (9)	0.0280 (8)	0.0019 (6)	0.0065 (6)	0.0038 (6)

### Geometric parameters (Å, °)

**Table:**

S1—C1	1.7407 (14)	C7—C8	1.517 (2)
S1—C4	1.7493 (14)	C7—H7A	0.9700
N1—C3	1.3471 (19)	C7—H7B	0.9700
N1—H1A	0.8600	C8—C9	1.523 (2)
N1—H1B	0.8600	C8—H8A	0.9700
N2—C6	1.147 (2)	C8—H8B	0.9700
N3—C5	1.149 (2)	C9—C10	1.522 (2)
N4—C1	1.3452 (19)	C9—H9A	0.9700
N4—C11	1.4710 (18)	C9—H9B	0.9700
N4—C7	1.4773 (18)	C10—C11	1.518 (2)
C1—C2	1.408 (2)	C10—H10A	0.9700
C2—C6	1.417 (2)	C10—H10B	0.9700
C2—C3	1.439 (2)	C11—H11A	0.9700
C3—C4	1.382 (2)	C11—H11B	0.9700
C4—C5	1.403 (2)		
			
C1—S1—C4	92.14 (7)	H7A—C7—H7B	107.9
C3—N1—H1A	120.0	C7—C8—C9	111.46 (14)
C3—N1—H1B	120.0	C7—C8—H8A	109.3
H1A—N1—H1B	120.0	C9—C8—H8A	109.3
C1—N4—C11	120.92 (12)	C7—C8—H8B	109.3
C1—N4—C7	118.17 (12)	C9—C8—H8B	109.3
C11—N4—C7	115.90 (12)	H8A—C8—H8B	108.0
N4—C1—C2	130.70 (13)	C10—C9—C8	109.21 (13)
N4—C1—S1	118.91 (10)	C10—C9—H9A	109.8
C2—C1—S1	110.39 (10)	C8—C9—H9A	109.8
C1—C2—C6	128.05 (13)	C10—C9—H9B	109.8
C1—C2—C3	113.45 (12)	C8—C9—H9B	109.8
C6—C2—C3	118.50 (13)	H9A—C9—H9B	108.3
N1—C3—C4	125.45 (13)	C11—C10—C9	111.98 (14)
N1—C3—C2	122.72 (13)	C11—C10—H10A	109.2
C4—C3—C2	111.82 (12)	C9—C10—H10A	109.2
C3—C4—C5	125.50 (13)	C11—C10—H10B	109.2
C3—C4—S1	112.16 (11)	C9—C10—H10B	109.2
C5—C4—S1	121.67 (11)	H10A—C10—H10B	107.9
N3—C5—C4	178.05 (15)	N4—C11—C10	111.79 (12)
N2—C6—C2	174.32 (16)	N4—C11—H11A	109.3
N4—C7—C8	111.97 (13)	C10—C11—H11A	109.3
N4—C7—H7A	109.2	N4—C11—H11B	109.3
C8—C7—H7A	109.2	C10—C11—H11B	109.3
N4—C7—H7B	109.2	H11A—C11—H11B	107.9
C8—C7—H7B	109.2		

### Hydrogen-bond geometry (Å, °)

**Table:**

*D*—H···*A*	*D*—H	H···*A*	*D*···*A*	*D*—H···*A*
N1—H1A···N3^i^	0.86	2.22	3.0576 (19)	164
N1—H1B···N2^ii^	0.86	2.29	3.0293 (17)	145
C7—H7A···S1	0.97	2.42	2.9041 (16)	110

Symmetry codes: (i) −*x*−1/2, −*y*+1/2, −*z*+1; (ii) −*x*, *y*, −*z*+1/2.
